# Development and Validation of the Eating Support for Healthcare Aides (ESHA) Questionnaire in Long-Term Care

**DOI:** 10.3390/nu17203235

**Published:** 2025-10-15

**Authors:** Chia-Hui Lin, Ming-Yi Liu

**Affiliations:** 1 Department of Nursing, Chang Gung University of Science and Technology, Chiayi Campus, Chiayi 61363, Taiwan; linchiahui@mail.cgust.edu.tw; 2 Center for Smart Healthcare Education, Chiayi Campus, Chiayi 61363, Taiwan; 3 Department of Senior Welfare and Services, Southern Taiwan University of Science and Technology, Tainan City 71005, Taiwan

**Keywords:** dysphagia, nutrition, eating support, healthcare aides, long-term care, food texture modification, questionnaire validation

## Abstract

**Background:** Swallowing difficulties (dysphagia) are highly prevalent among older adults and significantly contribute to malnutrition, dehydration, and poor health outcomes. Healthcare aides (HCAs), as frontline caregivers in long-term care, play a pivotal role in supporting residents’ nutritional intake. However, validated tools to evaluate their competencies in nutrition-related eating support are lacking. **Methods**: A cross-sectional study was conducted to develop and validate a competency-based questionnaire assessing healthcare aides’ knowledge, attitudes, and behaviors toward nutrition-focused eating support. Core domains, including oral function care, safe feeding practices, food texture modification, and nutrition safety, were identified through a systematic literature review and refined using a two-round modified Delphi process involving 26 experts. A 47-item questionnaire was then administered to 202 HCAs in Taiwan. Psychometric testing included item analysis, KR-20, Cronbach’s α, confirmatory factor analysis (CFA), composite reliability (CR), and average variance extracted (AVE). **Results**: The final instrument demonstrated strong content validity. The knowledge domain achieved acceptable reliability (KR-20 = 0.61), while the attitude and behavior domains showed excellent internal consistency (Cronbach’s α = 0.98). CFA confirmed good structural validity (χ^2^/*df* = 3.86, CFI = 0.93). CR and AVE values further supported construct validity. **Conclusions**: This nutrition-centered questionnaire is a valid and reliable tool to assess HCAs’ competencies in providing eating support. It offers a foundation for identifying training needs and designing educational programs aimed at preventing malnutrition and enhancing person-centered mealtime care in long-term care facilities.

## 1. Introduction

Swallowing difficulties, or dysphagia, are highly prevalent among older adults and individuals with physical or cognitive impairments. Approximately 30–40% of community-dwelling older adults experience some degree of dysphagia, with prevalence exceeding 60% among nursing home residents [1]. In populations with neurological or developmental disabilities, prevalence may reach up to 80% [2]. Dysphagia is associated with serious health consequences, including malnutrition, dehydration, aspiration pneumonia, and increased mortality risk [3]. Despite its high prevalence, dysphagia is often underdiagnosed and undertreated due to nonspecific symptoms, limited awareness, and inadequate access to specialized care services [4]. Feeding and mealtime assistance are therefore fundamental components of long-term care, directly influencing nutritional status, psychological well-being, and overall quality of life [5]. However, inadequate oral hygiene, improper feeding posture, failure to assess alertness, and inappropriate food textures can exacerbate dysphagia-related complications [6,7,8]. Although screening tools such as the Eating Assessment Tool (EAT-10) are available, their implementation is frequently hindered by insufficient caregiver training [9].

Healthcare aides (HCAs), who provide frontline care in institutional settings, play a pivotal role in supporting safe eating and nutrition. Unlike nurses or speech-language pathologists, HCAs often lack structured training in dysphagia management and person-centered feeding strategies [10]. Their caregiving practices are shaped by their knowledge, attitudes toward aging, beliefs about feeding autonomy, and organizational constraints. The absence of validated instruments for assessing HCAs’ competencies in this area has limited the effectiveness of training. In Japan, Kaigo Shokushi (介護食士, eating support for healthcare aides) play a pivotal role in long-term care by tailoring meals to the functional needs of older adults, addressing nutritional risks, ensuring food safety, and thereby contributing to improved health outcomes and quality of life [11]. While research on this role remains limited, it provides a culturally relevant model for structuring feeding-related competencies [7,8,12,13].

Most studies on dysphagia-related knowledge, attitudes, practices (KAP), or skills have focused on healthcare professionals working in neurology or surgical wards, particularly in relation to stroke-induced swallowing difficulties [4]. Recent literature also emphasizes the importance of nutrition knowledge and literacy in shaping dietary behaviors and guiding educational interventions [14,15,16,17].

Swallowing function is significantly affected by aging and oral health, yet these factors are often underrepresented in dysphagia management. Current literature highlights gaps in healthcare professionals’ knowledge and skills related to dysphagia, and recommends the integration of targeted education into nursing, nutrition, and speech-language curricula, with particular emphasis on nutrition strategies, food preparation, and safe feeding practices [18,19]. These findings highlight the need for inter-professional training and oral health education to improve patient safety and nutritional outcomes. To systematically define and measure these competencies, this study applies the Knowledge, Attitude, and Practice (KAP) framework, which explores the relationship between what individuals know, how they feel, and how they act. KAP-based tools—often referred to as Knowledge, Attitudes, and Behaviors (KAB) questionnaires—have been widely used to assess clinical decision-making and to design targeted educational interventions [19,20,21]. Guided by this framework, the present study aimed to develop and psychometrically validate a KAB questionnaire specifically designed to evaluate the competencies of healthcare aides in providing eating support. This tool is expected to guide caregiver training, foster person-centered mealtime care, and enhance the safety and quality of long-term care for older adults and individuals with swallowing difficulties.

## 2. Materials and Methods

### 2.1. Participants

Convenience sampling was used for this study. The inclusion criteria were as follows: individuals aged 20 years or older, certified as a home care service provider, and with at least one year of clinical experience. Individuals who were unwilling to participate or unable to read or write were excluded. Data were collected using paper-based questionnaires between 1 August 2024 and 31 January 2025. Based on previous recommendations, a minimum of 200 participants was required to conduct a confirmatory factor analysis [22], and this criterion was met in the present study.

### 2.2. Data Collection

Data were collected using a cross-sectional design with snowball sampling at community-based home care units and nursing home institutions. Recruitment and data collection were conducted in the southern and central regions of Taiwan, specifically in Nantou, Yunlin, Chiayi, and Tainan. The principal investigator provided a standardized explanation of the study’s purpose and procedures to potential participants. Each participant completed a paper-based questionnaire, which required approximately 20–30 min to complete. A total of 202 questionnaires were distributed, and all 202 were returned, yielding a 100% response rate.

All collected data were anonymized and stored in a password-protected digital file accessible only to the research team. Physical copies of the questionnaires were stored in a locked cable. The study adhered to strict confidentiality protocols, and no identifying information was recorded to ensure participant privacy.

### 2.3. Procedure

This study was conducted in two phases. Phase one focused on identifying the core professional competencies required of healthcare aides to provide eating support. Phase two involved the development of a measurement instrument to assess knowledge, attitudes, and behaviors aligned with these competencies, followed by an evaluation of its psychometric properties.

#### 2.3.1. Phase 1: Identification of Core Competencies

Based on the foundational work of Aziz and Campbell-Taylor, the care of individuals with swallowing difficulties requires specialized professional training and skills [10]. This study draws on the concept of the Kaigo Shoku-shi (介護食士) originating in Japan, where limited research has been con-ducted on its role and competencies. Building on this foundation, the present study defines the core competencies of Eating Support Healthcare Aides (ESHAs) in Taiwan as encompassing oral function care (e.g., oral hygiene and exercises), safe feeding practices, and food texture modification.

A systematic literature review was first conducted to establish a culturally relevant competency framework. Subsequently, a two-round modified Delphi technique was employed to refine and validate the framework. The modification involved using structured online surveys and providing summarized feedback between rounds, while maintaining the core principles of the traditional Delphi method, including iterative consensus-building and expert anonymity [23]. A total of 26 experts participated in the Delphi study. They included five professors of nutrition, four professors of oral hygiene, two professors of speech and swallowing therapy, two professors of nursing, two professors with expertise in core competencies, and eleven professors from the community long-term care field. Experts were purposively recruited based on their professional experience in practice or teaching within their respective fields. The average practical or teaching experience among the experts was 12 years (SD = 5.25), indicating substantial experience and high professional expertise. All experts provided informed consent prior to participation [24]. Through expert consensus, five major competency domains were identified: nutritional knowledge, food safety, oral function training, dietary modification, safe feeding techniques, and individualized care skills. These were further organized into four key responsibilities: (1) conducting chewing and swallowing screenings, (2) supporting clients in improving oral function and safe eating, (3) applying appropriate food textures tailored to individual needs, and (4) preparing and verifying meals according to personal requirements. This framework guided the development of the questionnaire, which initially included 31 items on knowledge, 12 items on attitude, and 12 items on behavior. See Supplement S1 for the Core Competencies.

#### 2.3.2. Phase 2: Tool Construction and Reliability Testing for Assessing Knowledge, Attitudes, and Behaviors in Eating Support Healthcare Aides

In the second phase, a competency-based instrument was developed and validated to assess knowledge, attitudes, and behaviors relevant to the role of Eating Support Healthcare Aides (ESHAs). Based on the established core competencies, 47 items were initially constructed. The knowledge section consisted of multiple-choice items derived from 31 core professional competencies, scored as either correct (1) or incorrect (0), with higher total scores indicating greater knowledge proficiency. The attitude and behavior sections included 10 and 12 items, respectively, and were rated on a five-point Likert scale ranging from 1 (strongly disagree) to 5 (strongly agree), with higher scores reflecting more positive attitudes and appropriate behaviors.

Three domain experts, including a registered nurse, a dietitian, and a speech-language pathologist, each with over ten years of clinical experience, were invited to evaluate the content validity of the instrument. Experts rated each item on a four-point scale with 1 representing not relevant, 2 somewhat relevant, 3 quite relevant, and 4 highly relevant. The item-level content validity index (I-CVI) was calculated as the proportion of experts rating an item as 3 or 4, and the scale-level content validity index (S-CVI) was calculated as the average of all I-CVIs. Items with I-CVI less than 0.78 were reviewed and revised based on expert feedback [25].

Item analysis was performed for the knowledge domain to examine the psychometric properties of individual items, including item difficulty (proportion of correct responses) and discrimination (point-biserial correlation with total score). Internal consistency of the knowledge items was assessed using the Kuder-Richardson Formula 20 (KR-20) [26], which reflects the degree to which items measure the same construct. Internal consistency of the attitude and behavior domains was evaluated using Cronbach’s alpha.

Construct validity was assessed through confirmatory factor analysis (CFA) with maximum likelihood estimation. A two-factor model was specified based on the theoretical structure of the instrument. Model fit was evaluated using multiple indices, including the chi-square to degrees of freedom ratio (χ^2^/*df*), normed fit index (NFI), incremental fit index (IFI), relative fit index (RFI), and comparative fit index (CFI) [27,28]. Convergent validity was further assessed by calculating composite reliability (CR) and average variance extracted (AVE) for each factor [29].

Demographic variables collected from the 202 participants included age, sex, education level, marital status, current employment in long-term care, years of work experience in long-term care, experience in caring for clients with swallowing or chewing difficulties, and possession of a Cook Class C certification. Age was recorded as a continuous variable (years). Sex was categorized as male or female. Education level was classified into six categories: elementary school, junior high school, senior/vocational high school, junior college/associate degree, bachelor’s degree, and master’s degree. Marital status was classified as married (widowed), married (spouse living), single (cohabiting), never married, or separated/divorced. Current employment in long-term care was categorized as day care, home care services, long-term care facility, home-based family care, community care center, or hospital. Work experience related to long-term care was measured in years. Experience in caring for clients with swallowing or chewing difficulties and possession of a Cook Class C certification were both recorded as yes/no variables.

### 2.4. Ethical Considerations

This study included experimental procedures that were reviewed by the Ethics Committee of the Chang Gung Medical Foundation Institutional Review Board (IRB No. 202302149B0). Informed consent was obtained from all participants. All methods and procedures used in this study were in accordance with the relevant guidelines and the Declaration of Helsinki. This cross-sectional study was conducted in accordance with the Strengthening the Reporting of Observational Studies in Epidemiology (STROBE) Statement [30]. The STROBE checklist was used to ensure that all key elements of the study design, participant selection, data collection, and analysis were transparently reported.

### 2.5. Data Analysis

Absolute (N) and relative (%) frequencies were used to describe categorical demographic variables, including age, sex, education level, marital status, current employment in long-term care, work experience related to long-term care, experience in caring for clients with swallowing or chewing difficulties, and possession of a Cook Class C Certification. Mean values and standard deviations (SD) were calculated for continuous variables. For the knowledge domain, item analysis and the Kuder-Richardson Formula 20 (KR-20) were used to assess internal consistency. The KR-20 coefficient was calculated using the following formula:

$$\text{KR-}20=\frac{k}{k-1}\left[1-\frac{\sum p_{i}q_{i}}{\sigma^{2}}\right]$$

where k represents the total number of items, $p_{i}$ is the proportion of participants who answered item i correctly, $q_{i}$ = 1 − $p_{i}$ is the proportion of participants who answered item i incorrectly, and σ^2^ is the variance of the total test scores. This formula is applied to dichotomous items (e.g., correct/incorrect responses) to assess internal consistency reliability. KR-20 values are commonly interpreted using three reliability levels: values below 0.50 are considered low, values between 0.50 and 0.80 are regarded as moderate [26,31].

The Difficulty Index (DI) for each item was calculated as the proportion of participants who answered the item correctly. Items with a DI between 0.30 and 0.80 were considered to have appropriate difficulty, while items with a DI below 0.30 or above 0.80 were regarded as too difficult or too easy, respectively. Item discrimination was simultaneously evaluated using the point-biserial correlation coefficient ($r_{p}b$) between each item and the total score, with $r_{p}b$ values interpreted as follows: <0.20 = low discrimination, 0.20–0.30 = moderate, and >0.30 = good [32].

For the attitude and behavior domains, internal consistency was evaluated using Cronbach’s alpha, with values of 0.70 or greater indicating acceptable reliability and values above 0.90 indicating excellent reliability [33]. Content validity was assessed using the item-level content validity index (I-CVI) and scale-level content validity index (S-CVI), with values of 0.78 or higher considered acceptable for expert agreement [25]. Three domain experts, including a registered nurse, a dietitian, and a speech-language pathologist, each with over ten years of clinical experience, rated the relevance of each item on a 4-point scale (1 = not relevant, 2 = somewhat relevant, 3 = quite relevant, 4 = highly relevant). Items with I-CVI below 0.78 were reviewed and revised based on expert feedback. Construct validity of the attitude and behavior domains was examined using confirmatory factor analysis (CFA) performed with AMOS version 24.0 using maximum likelihood estimation. A two-factor model was specified based on the theoretical structure of the instrument. Model fit was evaluated using the chi-square to degrees of freedom ratio (χ^2^/*df*), normed fit index (NFI), incremental fit index (IFI), relative fit index (RFI), and comparative fit index (CFI), with commonly accepted thresholds of χ^2^/*df* < 5.0, and NFI, IFI, RFI, CFI ≥ 0.90 [27,28]. Convergent validity was further assessed by calculating composite reliability (CR) and average variance extracted (AVE), with CR ≥ 0.70 and AVE ≥ 0.50 considered satisfactory, and AVE ≥ 0.36 regarded as acceptable according to Fornell and Larcker [28,29].

Statistical analyses, including item analysis, KR-20, *t*-tests, and CFA, were performed using IBM SPSS software version 26.0 and AMOS version 24.0, with significance set at *p* < 0.05.

## 3. Results

### 3.1. Sample Characteristics

Among the 202 participants, the majority were female (85.6%) with a mean age of 45.65 years (SD = 10.98). Most participants were married, lived with their spouses (54.5%), and had completed senior or vocational high school (37.6%). Regarding employment, the largest group worked in home care services (60.9%), with an average of 3.75 years (SD = 4.26) of experience in long-term care. Furthermore, 65.8% had experience in caring for clients with swallowing or chewing difficulties, whereas only 11.9% held a Class C Cook Certification. Table 1 presents the sample characteristics.

### 3.2. Content Validity

To evaluate the content validity of the questionnaire, a panel of three domain experts, including a registered nurse, a dietitian, and a speech-language pathologist, was invited to independently assess the relevance and clarity of each item across three domains: knowledge (25 items), attitude (10 items), and behavior (12 items). The Content Validity Index, calculated at both the I-CVI and S-CVI with all values exceeding 0.90.

### 3.3. Item Analysis and Reliability—Knowledge Domain

Item analysis was conducted to examine the psychometric properties of the 25-item knowledge scale. Higher scores on this scale correspond to a greater proportion of correct responses. The internal consistency of the scale was assessed using the KR-20, which is appropriate for dichotomously scored items and reflects the degree to which items measure the same construct. Given that this study employed a cross-sectional design without repeated measurements, KR-20 was used solely to assess internal consistency. The overall KR-20 coefficient was 0.61, which is considered modest but understandable given the multidimensional nature of the scale. The item difficulty indices ranged from 0.60 to 0.99. The point-biserial correlations ($r_{P}b$) for all 25 items ranged from 0.02 to 0.47. Notably, item 5 ($r_{p}b$ = 0.16), item 7 ($r_{P}b$ = 0.16), item 10 (rpb = 0.14), item 12 ($r_{p}b$ = 0.15), and 15 ($r_{p}b$ = 0.02) demonstrated insufficient discriminative power, indicating that these items were less effective in distinguishing between high- and low-performing respondents [32] (Table 2).

The finalized KAB questionnaire for Eating Support Healthcare Aides assessed core competencies in knowledge, attitudes, and behaviors related to feeding and mealtime care. The knowledge domain covered oral health, swallowing and chewing difficulties, safe feeding practices, food texture standards, hygiene, and nutrition. Full questionnaire items are provided in Supplement S2.

### 3.4. Confirmatory Factor Analysis

**Figure 1:**
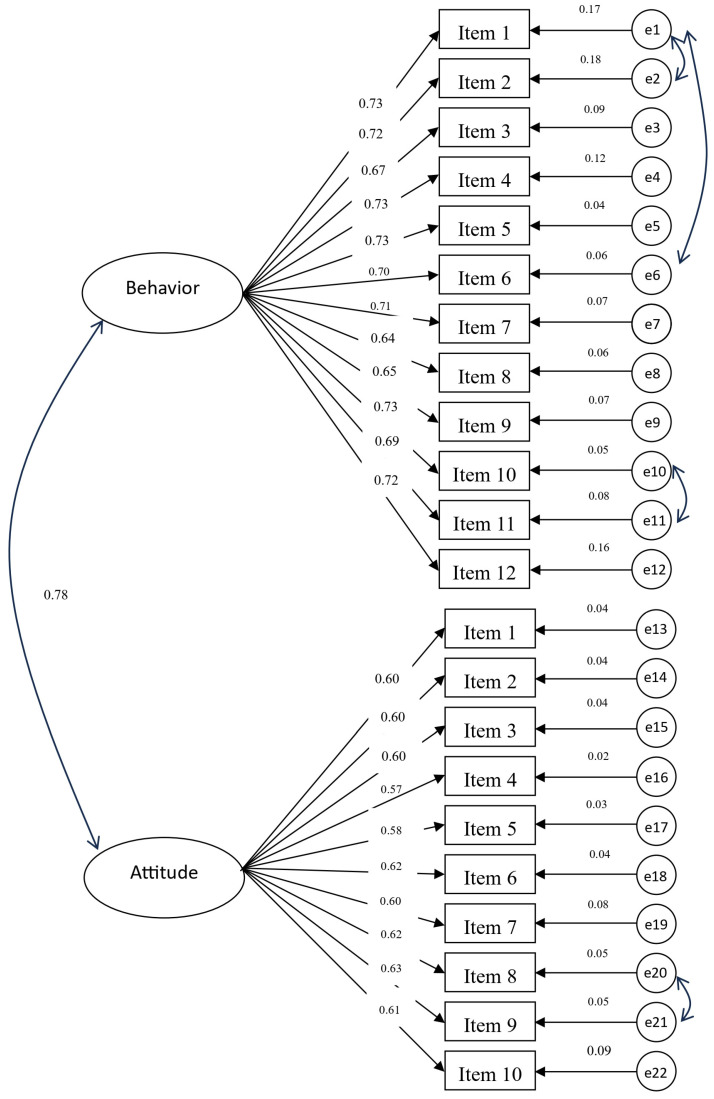
Confirmatory Factor Analysis results. Standardized path coefficients are shown. Solid arrows represent significant standardized paths, double-headed arrows indicate error covariances, and dashed arrows show modification index–suggested paths. Each observed variable has an associated error term (e1–e22), representing unexplained variance not accounted for by the latent constructs.

The knowledge domain of the ESHAs utilized dichotomous true-or-false response items, which are nominal variables and, therefore, not suitable for CFA. Consequently, CFA was not conducted on the knowledge component. Figure 1 presents the CFA results for the attitude and behavior components of ESHAs. The two-factor model demonstrated a good fit for the data. Specifically, the chi-square to degrees of freedom ratio (χ^2^*/df)* was 3.86, with fit indices indicating acceptable model adequacy: normed-fit index (NFI) = 0.91, incremental fit index (IFI) = 0.93, relative fit index (RFI) = 0.90, and comparative fit index (CFI) = 0.93.

### 3.5. Reliability and Validity—Attitude Domain

The attitude domain evaluated professional qualities such as responsibility, attentiveness, honesty, and teamwork. Participants actively provide dietary care for service users, collaborating with team members, adapting meal textures to individual needs, and ensuring hygiene and safety. They maintain professional development in managing chewing and swallowing disorders, and all meal preparation processes are carefully planned, executed, and monitored.

The internal consistency reliability of the attitude domain, consisting of 10 items, was excellent, with a Cronbach’s α of 0.98. Item-level analysis revealed Cronbach’s α values ranging from 0.87 to 0.96 and factor loadings between 0.56 and 0.63. All corrected item-total correlations exceeded 0.30, demonstrating a good internal consistency. The CR values, where available, ranged from 0.85 and above, while the AVE values met or exceeded 0.36, which is an acceptable threshold according to Fornell and Larcker [28] (Table 3).

### 3.6. Reliability and Validity—Behavior Domain

The behavior domain assessed practical skills in feeding and mealtime care. Participants could perform oral assessments (EAT-10, OHAT), apply feeding techniques under professional guidance, lead or assist in oral exercises, and manage meal preparation, including adjusting textures, using thickeners, ensuring hygiene, and preparing easy-to-chew or gingiva-friendly foods. They were also able to communicate with care teams and family members to meet the nutritional needs of service users. The behavioral domain of the scale demonstrated excellent internal consistency, with a Cronbach’s alpha of 0.98. The item-total correlations ranged from 0.87 to 0.95, indicating that all items contributed meaningfully to the overall construction. CFA revealed acceptable factor loadings ranging from 0.64 to 0.75, supporting the dimensionality of the construct. Additionally, the CR was 0.92, and the AVE was 0.50, both of which met the acceptable thresholds (CR > 0.70; AVE ≥ 0.50), indicating good convergent validity (see Table 4).

## 4. Discussion

This study evaluated the psychometric properties of a newly developed questionnaire designed to assess healthcare aides’ knowledge, attitudes, and behaviors toward eating support in long-term care. The results demonstrated robust validity and reliability across domains, supporting the use of the Eating Support for Healthcare Aides (ESHA) questionnaire as a comprehensive tool in this field.

Analysis of the knowledge domain items indicated moderate internal consistency, as reflected by a KR-20 coefficient of 0.61. Item difficulty indices ranged from 0.60 to 0.99, suggesting that participants were generally familiar with the fundamental aspects of eating support [26]. Discrimination indices further confirmed that the scale was effective in differentiating respondents with varying knowledge levels. Although five items (items 5, 7, 10, 12, and 15) exhibited relatively low point-biserial correlations ($r_{p}b$), indicating insufficient discriminative power, these items were retained following internal committee discussion [32]. Each of these items corresponds to a specific knowledge competency, and the primary aim was to assess participants’ learning in that domain rather than to make retention decisions solely based on statistical indicators. While previous literature suggests that increasing the number of items may help stabilize reliability, adding too many knowledge items could increase respondent burden [33,34]. Therefore, in this study, the decision to retain these items was guided by the educational purpose of the scale and considerations of participant workload.

The attitude domain exhibited excellent reliability, with a Cronbach’s alpha of 0.98 [32]. Factor loadings ranged from 0.56 to 0.63, and corrected item-total correlations exceeded 0.30. Composite reliability values were consistently above 0.85, while the average variance extracted (AVE) reached or exceeded 0.36, indicating satisfactory convergent validity [28,29]. The behavioral domain also demonstrated excellent internal consistency (Cronbach’s alpha = 0.98). Item-total correlations ranged from 0.87 to 0.95, with confirmatory factor analysis supporting the dimensional structure of the scale. Factor loadings (0.64–0.75), composite reliability (0.92), and AVE (0.50) all confirmed the convergent validity of this domain [28,29]. Collectively, these findings establish the ESHA as a psychometrically sound instrument for assessing healthcare aides’ competencies in eating support.

Previous research has shown that healthcare professionals often report only moderate levels of knowledge regarding dysphagia management, with significant gaps in standardized training on key competencies such as food texture modification, use of assessment tools, feeding techniques, and specialized diets [35,36]. Attitudes and practices are strongly influenced by knowledge, highlighting the critical role of education in improving dysphagia care and preventing adverse outcomes such as aspiration pneumonia in post-stroke patients [4]. In this context, the ESHA questionnaire provides a valuable framework for identifying educational needs, monitoring changes in competencies, and evaluating the effectiveness of training programs. From a clinical perspective, the ESHA can be used to guide the design of targeted, person-centered mealtime care interventions in long-term care facilities. By identifying knowledge and practice gaps, administrators and educators can tailor training content to the specific needs of healthcare aides. Future research should explore the cross-cultural applicability of the ESHA and its potential to capture longitudinal associations between improvements in aides’ competencies and resident outcomes, including nutritional status, swallowing safety, and overall quality of care. Furthermore, expanding the knowledge domain with more challenging items or alternative response formats may enhance the discriminatory capacity of the scale, particularly in advanced training contexts.

### 4.1. Implications for Nutrition and Long-Term Care

Adequate nutrition is essential for maintaining health and functional independence, particularly among older adults in long-term care settings. This is consistent with evidence indicating that nutrition knowledge is an important determinant of food choices, dietary intake adequacy, and overall health outcomes in various populations [37,38]. The findings of this study have practical significance for improving the safety and quality of eating support, as well as for addressing common nutritional challenges in long-term care facilities. Protein-energy malnutrition (PEM) and dehydration are major risk factors that contribute to functional decline, weakened immunity, and increased incidence of pressure ulcers among institutionalized residents [39]. “Healthcare aides’ competencies in nutrition-related knowledge (e.g., food texture modification, use of thickeners), attitudes, and behaviors can be systematically assessed utilizing the ESHA questionnaire developed in this study, thereby identifying educational needs in preventing protein-energy malnutrition (PEM) and dehydration. This could contribute to reduce the risk of functional decline, weakened immunity, and increased incidence of pressure ulcers among institutionalized residents due to PEM and dehydration. This highlights the potential of the ESHA tool not only as an assessment instrument but also as a driver to promote the adoption of international standardized dietary practices in long-term care settings.

From a clinical practice perspective, the application of the ESHA tool extends beyond curriculum development and educational training. It can also serve as a pre- and post-intervention evaluation measure to objectively assess training effectiveness and learning outcomes. Moreover, the tool can be incorporated into quality monitoring systems as a quality indicator, enabling administrators to track HCAs’ competencies in nutritional support on a regular basis and integrate such measures into long-term care quality assessment frameworks.

### 4.2. Strengths, Limitations, and Recommendations

This study had several strengths. The questionnaire was developed through a rigorous, multi-phase process, incorporating a modified Delphi method to define core competencies and a thorough psycho-metric evaluation based on confirmatory factor analysis. The inclusion of three distinct domains (knowledge, attitudes, and behaviors) offers a comprehensive framework for assessing the multidimensional competencies of ESHAs. Moreover, strong content validity and internal consistency supported the reliability and utility of the instrument in educational and evaluative contexts.

However, this study has several limitations that should be acknowledged. First, the use of convenience and snowball sampling, restricted to southern and central Taiwan, may limit the generalizability of the findings. Second, the self-reported nature of attitude and behavioral components may be subject to social desirability bias. Third, although the knowledge section was scored objectively, including more scenario-based or application-level items could have enhanced its practical relevance. Cultural specificity is another limitation of this study. Additionally, the knowledge assessment tool was inherently multidimensional. Consequently, the overall KR-20 reliability of 0.61 is understandable. KR-20 measures the internal consistency of the entire instrument; however, on a multidimensional scale, items across different domains are not necessarily highly correlated, resulting in a lower overall reliability. While some items exhibited low point-biserial correlations, they were retained because each item corresponded to a specific knowledge competency, and the primary aim was to assess participants’ learning rather than to make retention decisions solely based on statistical indices. Future studies could improve reliability by increasing the number of items within each domain, refining item design, or conducting pilot testing, while carefully balancing participant burden to avoid excessive cognitive load. Competencies and questionnaire items were developed based on Taiwanese care practices, food culture, and regulatory norms. Therefore, cross-cultural adaptation and validation are recommended before applying this instrument to different regions or populations.

## 5. Conclusions

This study successfully developed and validated a questionnaire designed to assess healthcare aides’ knowledge, attitudes, and behaviors regarding eating support in long-term care settings. The instrument demonstrated clear content and construct validity, as well as acceptable item difficulty and discrimination indices. Although the overall internal consistency reliability was moderate, the questionnaire is grounded in a competency-based framework and provides a culturally relevant and evidence-informed approach to evaluate the core capabilities of eating support healthcare aides.

These findings support the use of this questionnaire as a reliable and valid instrument for assessing training needs, guiding the development of educational programs, and evaluating intervention out-comes. Since eating support is a critical component of person-centered care, particularly for older adults with swallowing or chewing difficulties, implementing this tool has the potential to enhance care quality and safety across various long-term care settings. Future research should examine the applicability of this approach in different populations and contexts and evaluate its responsiveness to changes following targeted training interventions.

## Figures and Tables

**Table 1:** Demographic characteristics (N = 202).

Variable	Characteristics	N	%
Sex	Male	29	14.4
	Female	173	85.6
Age(M ± SD), years	45.65 ± 10.98		
Marital status	Married (Widowed)	17	8.4
	Married (Spouse living)	110	54.5
	Single (Cohabiting)	2	1.0
	Never Married	39	19.3
	Separated or Divorced	34	16.8
Educational level	Elementary school	4	2.0
	Junior high school	26	12.9
	Senior high school/Vocational high school	76	37.6
	Junior college/Associate degree	37	18.3
	Bachelor’s degree	48	23.8
	Master’s degree	11	5.4
Currently employed in long-term care	Day Care/Small-Scale Multifunctional Services	13	6.4
	Home Care Services	123	60.9
	Care Facility/Long-Term Care Facility	37	18.3
	Home-Based Family Care	3	1.5
	Community Care Center (C Site)	5	2.5
	Hospital	21	10.4
Work Experience Related to Long-Term Care (M ± SD), years	3.75 ± 4.26		
Cared for clients with swallowing or chewing difficulties	Yes	133	65.8
	No	69	34.2
Cook Class C Certification	Yes	24	11.9
	No	178	88.1

Note. M = mean; SD = standard deviation.

**Table 2:** Item analysis of the knowledge domain, including difficulty indices, discrimination indices, and internal consistency (KR-20).

Item No.	Competency Code	Competency Description	KR-20	Difficulty Index	$r_{P}b$	Discrimination
1	K01	Understand the structure and function of oral chewing mechanisms.	0.61	0.96	0.30	Acceptable
2	K02	Recognize common chewing and swallowing difficulties in daily life.		0.92	0.30	Acceptable
3	K03	Understand the systemic health impacts of oral diseases.		0.93	0.30	Acceptable
4	K04	Identify high-risk groups for swallowing and chewing disorders.		0.83	0.30	Acceptable
5	K05	Understand the roles of interprofessional care teams.		0.96	0.16	insufficient discriminative power
6	K09	Prepare a safe and appropriate eating environment for care recipients.		0.94	0.38	Acceptable
7	K02	Identify incorrect statements about swallowing assessment tools.		0.93	0.16	insufficient discriminative power
8	K10, K11	Apply knowledge of eating assistive devices.		0.96	0.24	Acceptable
9	K12	Use multisensory stimulation techniques to enhance appetite.		0.91	0.40	Acceptable
10	K06	Conduct proper oral assessment and cleaning.		0.98	0.14	insufficient discriminative power
11	K06	Understand the Bass toothbrushing technique.		0.95	0.35	Acceptable
12	K07	Maintain and clean dentures correctly.		0.97	0.15	insufficient discriminative power
13	K06	Apply oral moisturizing products appropriately.		0.90	0.40	Acceptable
14	K06	Use alternative methods for oral care.		0.97	0.30	Acceptable
15	K08	Understand and implement oral exercises.		0.98	0.02	insufficient discriminative power
16	K13	Understand the IDDSI framework levels.		0.96	0.30	Acceptable
17	K14, K16	Test and evaluate food texture.		0.95	0.32	Acceptable
18	K17	Identify high-risk foods that may cause choking.		0.86	0.35	Acceptable
19	K18	Understand types and use of thickening agents.		0.93	0.30	Acceptable
20	K19, K20	Apply correct practices for food preparation and storage.		0.60	0.32	Acceptable
21	K19	Ensure food safety during reheating processes.		0.80	0.43	Acceptable
22	K21, K23	Prevent food poisoning using safe practices.		0.93	0.33	Acceptable
23	K25	Understand the dietary principle of ‘Three Goods and One Skill’.		0.96	0.30	Acceptable
24	K25	Identify tenderization techniques for food preparation.		0.61	0.47	Acceptable
25	K26, K27	Apply dietary management for dialysis-related conditions.		0.64	0.40	Acceptable

Notes: KR-20 = Kuder–Richardson Formula 20, used to evaluate the internal consistency reliability of the knowledge scale; Difficulty Index (DI) = proportion of participants who answered each item correctly; $r_{p}b$ = point-biserial correlation coefficient, used to assess each item’s discriminative power.

**Table 3:** Reliability and validity for the attitude domain.

Item No.	Competency Code	Item Description	Cronbach’s α	Factor Loading	CR	AVE
1	A01	I actively participate in the care of service users and proactively assist in resolving issues related to their dietary needs.	0.87	0.60	0.85	0.36
2	A02. A09	In my work, I maintain an honest and sincere attitude to protect the interests of the service users.	0.92	0.60		
3	A03	I care for service users with patience and attentiveness, respecting and understanding their needs related to chewing and swallowing.	0.92	0.60		
4	A04	I will continue to develop my professional competence in chewing and swallowing disorders, as well as in modifying food textures.	0.89	0.57		
5	A05	I take responsibility for my work and ensure that the meals I prepare meet hygiene and safety standards.	0.94	0.58		
6	A06, A09	I am willing to collaborate with dietitians and other team members to adjust food texture and nutritional content based on the health conditions of the care recipients.	0.94	0.62		
7	A07	When preparing meals, I handle food and ingredients with care to ensure that the texture is appropriate for the care recipients’ chewing and swallowing abilities.	0.96	0.60		
8	A08. A09. A10	I can adapt meal recipes flexibly based on the care recipients’ health conditions and daily needs.	0.94	0.62		
9	A11	I plan each meal’s ingredients in advance according to the individual needs of each care recipient and manage ingredient usage efficiently to avoid waste.	0.95	0.63		
10	A12	When preparing and distributing meals, I carry out each step precisely and continuously monitor the process to ensure compliance with safety and hygiene standards.	0.93	0.61		

Note. composite reliability (CR), average variance extracted (AVE).

**Table 4:** Reliability and Validity Indicators for the Behavior Domain Items.

Item No.	Competency Code	Item Description	Cronbach’s α	Factor Loading	CR	AVE
1	B01	I am able to accurately perform the simple screening for chewing and swallowing disorders (EAT-10).	0.87	0.73	0.92	0.50
2	B02	I am able to use the Oral Health Assessment Tool (OHAT).	0.87	0.72		
3	B03	I am able to select appropriate cleaning methods and assistive tools.	0.91	0.67		
4	B04	I can lead/assist in “Jia Bai Er Swallowing Exercise.”	0.90	0.73		
5	B05	I can select and use feeding techniques/tools under professional guidance.	0.95	0.73		
6	B06	I can measure and adjust the texture/quality of meals.	0.93	0.70		
7	B07	I can use thickeners appropriately.	0.92	0.71		
8	B08	I understand and perform proper cleaning/disinfection of utensils.	0.92	0.64		
9	B09	I can manage ingredients, assess freshness, and provide high-quality meals.	0.91	0.75		
10	B10	I can communicate with nutritionists/family to meet nutritional needs.	0.94	0.73		
11	B11	I can prepare meals based on ingredient characteristics and cooking techniques.	0.90	0.69		
12	B12	I can prepare easy-to-chew and gingiva-friendly minced soft foods.	0.88	0.72		

Note. composite reliability (CR), average variance extracted (AVE).

## Data Availability

The data are available from the corresponding author upon reasonable request due to privacy.

## Supplementary Materials

The following supporting information can be downloaded at: https://www.mdpi.com/article/10.3390/nu17203235/s1, Supplement S1: Supplement for the Eating Support Healthcare Aides Core Competencies; Supplement S2: Supplement for finalized Eating Support Healthcare Aides items.

## Abbreviations

The following abbreviations are used in this manuscript:

HCAs	Healthcare Aides
KABs	Knowledge, Attitudes, and Behaviors
KAP	Knowledge, Attitude, and Practice
CFA	Confirmatory Factor Analysis
CR	Composite Reliability
AVE	Average Variance Extracted
ESHAs	Eating Support Healthcare Aides
KR-20	Kuder Richardson Formula 20
SDs	Standard Deviations
M	Mean
I-CVI	Item Content Validity Index
S-CVI	Scale Content Validity Index
NFI	Normed-Fit Index
IFI	Incremental Fit Index
RFI	Relative Fit Index
CFI	Comparative Fit Index
