# Supplementary material for: Development and Validation of the Eating Support for Healthcare Aides (ESHA) Questionnaire in Long-Term Care
Source: Nutrients. 2025 Oct 15;17(20):3235. doi: 10.3390/nu17203235 (PMC12567063; doi:10.3390/nu17203235)
Supplement: Supplementary file 1 [file nutrients-17-03235-s001.zip › Supplement S1.pdf]

Supplement S1. Supplement for the Eating Support Healthcare Aides Core Competencies

Knowledge Core Competencies	Attitude Core Competencies	Behaviors Core Competencies
K01.Understanding the Structure and Mechanism of Oral Chewing	A01. Proactiveness	B01. Able to perform simple screening for chewing and swallowing disorders (EAT-10).
K02.Understanding Common Daily Phenomena of Swallowing and Chewing Difficulties	A02. Honesty and Sincerity	B02. Able to perform the Oral Health Assessment Tool (OHAT) evaluation.
K03.Understanding the Relationship Between Oral Diseases and Systemic Health	A03. Attentive Care	B03. Ability to assist with oral hygiene care, including selecting appropriate cleaning methods and aids.
K04. Physiological Conditions Affecting Swallowing and Chewing Functions in Care Recipients	A04. Continuous Learning	B04. Ability to lead or assist in performing oral exercises ("Jia Bai Er Swallowing Exercise") to promote oral health.
K05. Roles and Responsibilities of the Interprofessional Care Team in Managing Chewing and Swallowing Disorders	A05. Responsibility	B05. Able to provide correct feeding/eating techniques and select feeding tools under the guidance of professionals (e.g., speech therapists).
K06. Knowledge of Proper Oral Hygiene Practices	A06. Interdisciplinary Teamwork	B06. Skills in measuring the texture and quality of meals.
K07. Knowledge of Denture Cleaning and Maintenance	A07. Diligence and Meticulousness	B07. Skills in using thickeners.
K08. Understanding of Oral Health-Related Knowledge	A08. Flexibility	B08. Skills in cleaning and disinfecting utensils.
K09. Knowledge of Preparing a Safe Eating Environment and Equipment for Care Recipients	A09. Professionalism	B09. Ability to manage and assess the freshness of ingredients.
K10. Knowledge of Basic Feeding Assistance and Safe Eating Posture Preparation for Care Recipients	A10. Flexibility	B10. Able to communicate effectively with nutrition professionals or family members and provide nutritionally appropriate meals.
K11. Knowledge of Eating Assistive Devices and Their Application	A11. Organization and Planning	B11. Able to prepare meals that meet care recipients' needs based on ingredient characteristics and cooking techniques.
K12. Application of Multisensory	A12. Accurate	B12. Ability to prepare

Stimulation and Appetite Enhancement Methods	Execution and Monitoring	"easy-to-chew soft foods" and "gingiva-friendly minced soft foods."
K13. Explanation of the IDDSI (International Dysphagia Diet Standardisation Initiative) Framework Levels		
K14. Understanding Taiwan's Local Food Texture Classification Standards and Their Theoretical Basis		
K15. The measurement methods for food texture quality mentioned in the "Nutrition and Food Texture Education Manual for Older Adults" by the Ministry of Health and Welfare, Taiwan, and their correspondence with the international IDDSI framework.		
K16. Understanding Taiwan's Food Texture Testing Methods		
K17. Understanding the Characteristics and Potential Risks of Common Aspiration-Prone Foods		
K18. Understanding the Types, Characteristics, and Applications of Thickeners		
K19. Knowledge of Food Selection, Storage, Handling, and Management		
K20. Skills in Basic Food Preparation Procedures (e.g., Cutting, Washing)		
K21. Methods for Preventing Food Poisoning		
K22. Basic knowledge of hygiene in the cooking environment.		
K23. Basic Knowledge of Food Hygiene and Safety		
K24. Geriatric nutrition.		
K25. The Importance of a Balanced Diet, National Dietary Guidelines, and Major Nutrient Sources (Three Goods and One Smart Principle)		
K26. Physiological Changes and Their Nutritional Needs		
K27. Dietary Principles for		

Common Diseases		
