# Supplementary material for: Development and Validation of the Eating Support for Healthcare Aides (ESHA) Questionnaire in Long-Term Care
Source: Nutrients. 2025 Oct 15;17(20):3235. doi: 10.3390/nu17203235 (PMC12567063; doi:10.3390/nu17203235)
Supplement: Supplementary file 1 [file nutrients-17-03235-s001.zip › Supplement S2.pdf]

Supplement S2. Supplement for finalized Eating Support Healthcare Aides items.

Knowledge items
1. Regarding the structure and mechanism of oral mastication, which of the following is correct?<br>(A) Chewing mainly relies on the tongue to manipulate food, and teeth are not necessary.<br>(B) The chewing process has no direct relationship with the smoothness of swallowing.<br>(C) During chewing, oral muscles are not involved; it mainly depends on pharyngeal muscles.<br>(D) Chewing involves multiple oral structures working together, including teeth occlusion, lip closure, and tongue manipulation.<br>**Correct answer:** D
2. Which of the following is an incorrect description of common chewing and swallowing difficulties in daily life?<br>(A) Maintain an upright posture with a slightly tucked chin and a mild backward lean while eating.<br>(B) Choose appropriate food textures and use suitable adaptive utensils, such as assistive cups or spoons.<br>(C) Eat slowly without rushing the person.<br>(D) Encourage and teach the individual to perform active oral exercises.<br>**Correct answer:** A
3. Which of the following best explains the impact of oral diseases on overall health?<br>(A) Oral diseases are limited to dental problems and have no impact on overall health.<br>(B) Oral bacterial infections may increase the risk of cardiovascular diseases.<br>(C) Oral diseases only affect the digestive system and do not impact other body systems.<br>(D) Oral diseases only affect the mouth and do not cause systemic problems.<br>**Correct answer:** B
4. Which of the following is not a high-risk group for chewing and swallowing disorders?<br>(A) Patients with brain injuries (e.g., stroke), neurodegenerative diseases (e.g., Parkinson's disease, dementia), or head and neck cancers (e.g., oral cancer).<br>(B) Older adults<br>(C) Individuals with hypertension or hyperlipidemia<br>(D) Individuals who frequently cough or choke<br>**Correct answer:** C
5. Regarding the roles and responsibilities of the interdisciplinary care team for chewing and swallowing disorders, which statement is correct?<br>(A) Chewing and swallowing issues should be managed solely by a dietitian.<br>(B) Physicians, nurses, occupational therapists, and other professionals should collaborate to provide comprehensive care tailored to the individual's needs.<br>(C) Team members do not need to participate in chewing and swallowing care; only nurses are required.<br>(D) Chewing and swallowing problems do not require a multidisciplinary team and are usually adapted by the individual.<br>**Correct answer:** B
6. When preparing a safe eating environment for clients, which of the following is the correct practice?<br>(A) The dining environment does not require much consideration, mainly relying on the individual's eating skills.<br>(B) The mealtime environment can be simply arranged without special preparation.<br>(C) Ensure the dining environment is clean and safe, and prepare appropriate eating utensils

and stable objects.<br>(D) The preparation of eating materials can be left to the individual to enhance autonomy.<br>**Correct answer:** C
7. Which of the following statements about the Eat-10 swallowing assessment scale is incorrect?<br>(A) The EAT-10 is a self-assessment tool used to evaluate the severity of swallowing difficulties.<br>(B) It is a questionnaire that can only be completed by healthcare professionals.<br>(C) A total EAT-10 score of 3 or higher indicates a potential risk of swallowing difficulties.<br>(D) The EAT-10 consists of 10 questions, each rated on a 5-point scale (0, 1, 2, 3, 4).<br>**Correct answer:** B
8. Regarding knowledge and application of feeding aids, which statement is correct?<br>(A) The choice of aids does not affect eating safety, as long as the user adapts to them.<br>(B) Eating aids should be selected based on the user's needs, and proper instruction should be provided to enhance eating safety.<br>(C) Aids should be changed randomly to help the user adapt to different devices.<br>(D) Eating aids only need to be used when nursing staff are present.<br>**Correct Answer:** B
9. When promoting clients' appetite, which of the following is a correct multisensory stimulation method?<br>(A) Serve bland meals to avoid stimulation, keeping the eating environment quiet and comfortable.<br>(B) Reduce food choices to prevent picky eating.<br>(C) Provide a single type of food to aid digestion without adding sensory stimulation.<br>(D) Use colorful foods with appealing aromas and varied textures to increase the client's desire to eat.<br>**Correct Answer:** D
10. When conducting oral assessment and cleaning, which of the following is the correct practice?<br>(A) Examine the lips, teeth, gums, tongue, and other oral areas, using appropriate cleaning tools.<br>(B) Oral cleaning only needs to be done after the client eats.<br>(C) Any toothbrush can be used without considering the cleaning method.<br>(D) Oral assessment does not require defined scope or frequency, as long as cleaning is done regularly.<br>**Correct Answer:** A
11. Which of the following correctly describes the Bass toothbrushing method?<br>(A) Place the toothbrush on the tooth surface and brush horizontally.<br>(B) Use an electric toothbrush to brush quickly back and forth on the tooth surface.<br>(C) Only use a floss pick to clean between the teeth.<br>(D) Place the toothbrush at the gum-tooth junction at a 45-degree angle, using small circular motions to clean each tooth.<br>**Correct Answer:** D
12. Regarding denture cleaning and maintenance, which statement is correct?<br>(A) Dentures should be cleaned daily using a specialized cleanser to protect the material.<br>(B) Dentures do not need daily cleaning and can be cleaned once a week.<br>(C) Dentures should be worn at all times and do not require regular cleaning or maintenance.<br>(D) After cleaning, dentures can be soaked in hot water for sterilization.<br>**Correct Answer:** A

13. In the use of oral moisturizing products, which statement is correct?
(A) Oral moisturizing products are only suitable for use at night.
(B) Oral moisturizing products can be used regularly for individuals with dry mouth to maintain oral moisture.
(C) Oral moisturizing products only need to be applied to the gums.
(D) Oral moisturizing products should not be used long-term to avoid dependency.
Correct Answer: B

14. Regarding other oral care methods, which statement is correct?
(A) Fluoride application is only suitable for children, not adults.
(B) Regular oral examinations help detect oral problems early and reduce the risk of disease.
(C) Oral care does not require regular check-ups, only daily self-cleaning.
(D) Special oral care policies only apply to medical institutions, not individual care.
Correct Answer: B

15. Which of the following is correct about the functions of oral exercise (Kenkou Taiso)?
(A) Oral exercises are only suitable for young people; older adults should not perform them.
(B) Oral exercises can stimulate oral muscle activity and help maintain oral health.
(C) Oral exercises cannot improve oral function and are only for leisure.
(D) Oral exercises only need to be done occasionally and do not require regular practice.
Correct Answer: B

16. Regarding the International Dysphagia Diet Standardization Initiative (IDDSI) levels, which statement is correct?
(A) IDDSI standards are only applicable to hospitalized patients and do not need widespread use.
(B) IDDSI levels help clearly distinguish different food textures, reducing the risk of choking in individuals with swallowing difficulties.
(C) IDDSI levels apply only to solid foods; beverages do not need classification.
(D) IDDSI is only applicable to children and older adults, not other populations.
Correct Answer: B

17. When testing food texture, which statement is correct?
(A) Use fingers to test the food's hardness.
(B) Bite the food directly with teeth to test hardness.
(C) Observe the color of the food to determine its texture.
(D) Press the food with a knife, fork, or spoon to observe deformation and ease of chewing.
Correct Answer: D

18. Which of the following foods may cause choking and are considered high-risk?
(A) Soft cooked rice porridge
(B) Soft and mushy ingredients
(C) Fruit puree
(D) Hard rice crackers
Correct Answer: D

19. Regarding the types and uses of thickening agents, which statement is correct?
(A) Thickened liquids may remain in the pharynx of people with dysphagia, increasing the risk of choking.
(B) Thickeners can be added arbitrarily, as long as the liquid becomes thicker.
(C) The main function of thickeners is to strengthen swallowing muscles and help dysphagic individuals regain swallowing ability.
(D) Liquids thickened with thickeners can be stored for several days for later use.

**Correct Answer:** A
20. Regarding food preparation and storage, which statement is correct?<br>(A) Meat should be thawed on a clean countertop at room temperature.<br>(B) Ensure ingredients are fresh and follow the first-in, first-out principle to reduce food waste.<br>(C) Food preprocessing involves removing inedible parts, such as fruit seeds, peels, bones in meat, or tougher parts of ingredients.<br>(D) Apples for direct blending should be prepared using a raw food knife.<br>**Correct Answer:** C
21. When reheating refrigerated or frozen dishes, which rule should be followed to ensure food safety?<br>(A) Reheating can be done unlimited times, but the temperature must reach above 75 °C.<br>(B) Reheating should be limited to once, and the core temperature must reach above 75 °C.<br>(C) Reheating can be done unlimited times, but the temperature must reach above 100 °C.<br>(D) Reheating can be done twice, and the core temperature must reach above 75 °C.<br>**Correct Answer:** B
22. Which of the following is a correct method to prevent food poisoning?<br>(A) The “Five Do’s and Two Don’ts” rule: wash hands, use fresh ingredients, separate raw and cooked foods, heat thoroughly, pay attention to storage temperature, do not drink spring water, do not consume unknown plants or animals.<br>(B) Cooked food must reach a temperature of 45°C or above to kill pathogens such as *Vibrio parahaemolyticus*, *Staphylococcus aureus*, *Bacillus cereus*, and *Salmonella* spp.<br>(C) Allergenic and non-allergenic ingredients should be handled separately, and allergenic ingredients should be processed first.<br>(D) Utensils that have been washed or disinfected can be wiped with a cloth.<br>**Correct Answer**: A
23. According to the “Three Goods and One Skill” dietary principle, which description is correct?<br>(A) “Able to eat” refers to considering the elderly person’s food preferences—just being able to eat is sufficient.<br>(B) “Eat correctly” refers to consuming the six major food groups; fruits can replace vegetables to avoid insufficient fiber.<br>(C) “Eat enough” means using small, frequent meals to obtain sufficient daily calories and nutrients.<br>(D) “Eat smart” refers to adding large amounts of oil, sugar, and salt during cooking to enhance flavor and nutrition.<br>**Correct Answer:** C
24. Regarding methods to make meat tender, which statement is incorrect?<br>(A) Marinating meat with fruits like pineapple or kiwi helps tenderize the meat.<br>(B) Scoring the meat can shorten cooking time and allow the flavor to penetrate.<br>(C) Cooking meat quickly at high temperatures can make it tender.<br>(D) Sprinkling salt helps retain moisture in meat, improves protein structure, and enhances tenderness.<br>**Correct Answer**: C
25. Regarding dietary management principles for common kidney disease, which statement is correct?<br>(A) Foods contain water—fruits about 90%, meat about 60%, and starchy foods (cooked rice and noodles) about 60%—and these should be included in the calculation of daily water intake.<br>(B) The fluid intake for dialysis patients should be based on the patient’s urine output plus

100 mL.<br>(C) Choose low-potassium, water-rich fruits and vegetables, such as melons, strawberries, and kiwi.<br>(D) Take medications separately whenever possible, which increases the amount of water consumed with medicine.<br>**Correct Answer**: A					
Attitudes<br>Please indicate the extent to which you agree or disagree with each statement by selecting one of the following options on a 5-point Likert scale:	Strongly agree	Agree	Neutral	Disagree	Strongly disagree
1.I will actively participate in the care of service users and assist in resolving meal-related issues.	5	4	3	2	1
2.In my work, I will maintain integrity and sincerity to protect the interests of service users.	5	4	3	2	1
3. I will provide patient and attentive care, respecting and understanding the chewing and swallowing needs of service users.	5	4	3	2	1
4. I will continuously learn professional knowledge and skills related to chewing and swallowing disorders and food texture modification to enhance my competence.	5	4	3	2	1
5. I will take responsibility for my work and ensure that prepared meals meet hygiene and safety standards.	5	4	3	2	1
6. I am willing to collaborate with dietitians and other team members to adjust meal texture and nutrition according to the health conditions of service users.	5	4	3	2	1
7. I will handle meal preparation and food processing carefully to ensure the texture matches the chewing and swallowing abilities of service users.	5	4	3	2	1
8.I can flexibly adjust meal recipes according to the health conditions and daily needs of service users.	5	4	3	2	1
9.I will plan each meal in advance based on individual needs and manage ingredients efficiently to avoid waste.	5	4	3	2	1
10. I will execute and monitor meal preparation and distribution precisely to ensure safety and hygiene	5	4	3	2	1

requirements are met.					
Behaviors<br>Please indicate the extent to which you agree or disagree with each statement by selecting one of the following options on a 5-point Likert scale:	Strongly agree	Agree	Neutral	Disagree	Strongly disagree
1.I am able to correctly administer a simple screening for chewing and swallowing disorders (EAT-10) to assess the safety of eating.	5	4	3	2	1
2. I can use the Oral Health Assessment Tool (OHAT) to evaluate the oral health status of service users.	5	4	3	2	1
3. I can select appropriate cleaning methods and assistive tools to help service users maintain good oral hygiene.	5	4	3	2	1
4. I can lead or assist service users in performing the "Eat Swallowing Health Exercise" to promote oral and swallowing health.	5	4	3	2	1
5. I am able to correctly select and apply feeding techniques and tools under the guidance of professionals (e.g., speech therapists).	5	4	3	2	1
6. I possess the skills to measure and adjust food texture quality to meet the chewing and swallowing needs of service users.	5	4	3	2	1
7. I can correctly use thickening agents to modify food texture according to service users' needs.	5	4	3	2	1
8. I understand the procedures for cleaning and disinfecting tableware and can carry them out correctly to ensure hygiene.	5	4	3	2	1
9. I can effectively manage food ingredients, assess their freshness, and provide high-quality meals.	5	4	3	2	1
10. I can communicate effectively with nutrition professionals or family members to provide meals that meet the nutritional needs of service users.	5	4	3	2	1
11. I can prepare meals that meet service users' needs by applying knowledge of ingredient characteristics and cooking techniques.	5	4	3	2	1

12. I am capable of preparing “easy-to-chew soft foods” and “gums-fragmented soft foods” to match the chewing and swallowing abilities of service users.	5	4	3	2	1
